# Bacterial profile and antimicrobial susceptibility patterns of otitis media in Ayder Teaching and Referral Hospital, Mekelle University, Northern Ethiopia

**DOI:** 10.1186/s40064-015-1471-z

**Published:** 2015-11-14

**Authors:** Araya Gebereyesus Wasihun, Yilikal Zemene

**Affiliations:** Department of Medical Microbiology and Immunology, Institute of Biomedical Sciences, College of Health Sciences, Mekelle University, Mekelle, Ethiopia; Department of ENT, School of Medicine, College of Health Sciences, Mekelle University, Mekelle, Ethiopia

**Keywords:** Otitis media, Bacterial isolates, Drug susceptibility, ENT, Ayder Referral Hospital

## Abstract

Middle Ear infection is a common problem for both children and adults particularly in resource limited countries. Nevertheless, in Ethiopia and particularly in the study area, there is scarcity of recent data that indicate the magnitude of the problem. Thus this study aimed to identify bacterial isolates and determine their drug susceptibility patterns from patients who had ear infection. Cross sectional study was carried out on patients with ear infection and who visited the Ear, Nose and Throat clinic of Ayder referral and teaching hospital from November 2014 to June 2015. Middle ear discharges were collected and processed for bacterial culture and antimicrobial susceptibility testing using standard bacteriological techniques. Clinical and demographic data were collected using standard questionnaire. Data were entered and analyzed using SPSS version 20 software and p value of < 0.05 was considered statistically significant. Of the total of 162 patients with ear discharges, 68.5 % were from rural areas, 71 % with chronic infection, 54.9 % referred cases and 67.3 % of them had decreased hearing status. Pathogens were isolated from 157 (98.2 %) of the patients with a total of 216 isolates. *Staphylococcus aureus* 46 (28.4 %), *Proteus mirabilis* 39 (24.1 %), *Pseudomonas aeruginosa* 27 (16.7 %), *Klebsiella* spp. and *Haemophilus influenzae* 18 (11.1 % each) were the dominant bacteria. Out of the individuals with ear infection, single and mixed bacterial infection was seen among 185 (90.7 %) and 59 (39.5 %) respectively. Age group of 0–5 years (p = 0.02), chronic patients (p = 0.042) and referred cases (p = 0.045) showed high bacterial isolates. High resistance was seen to most antibiotics. Ciprofloxacin, Gentamicin Norfloxacin and Erythromycin were effective against isolated bacteria. The overall multi drug resistance rate of bacteria in this study was 74.5 %. Prevalence of bacteria associated with otitis media and multidrug resistance was very high in the study area. Ciprofloxacin, gentamicin, norfloxacin and erythromycin can be used to treat otitis media. Treatment of patients should be based on antimicrobial susceptibility test to prevent complications, development of further antibiotic resistance and extra treatment costs.

## Background

Otitis media (OM), an inflammation of the middle ear cleft, is a common problem worldwide (Cripps and Kyd 2003). Globally, about 65–330 million people suffer from ear infection and 60 % of them had significant hearing impairment (Woodfield and Dugdale 2008). If left untreated, OM leads to more complications including recurrent acute otitis media, persistence of middle ear effusion which requires the insertion of drainage tube, hearing impairment, mastoiditis, meningitis, chronic otitis media, brain abscess and sepsis (Winn et al. 2006). Due to the low socio-economic status, overcrowding, poor hygiene, inadequate health care, and recurrent upper respiratory tract infection, the burden is high in low and middle income countries (Kumar and Seth 2011; Akinpelu et al. 2008).

Although ear infection is a common problem for all age groups (Bluestone and Klein 2001), due to the shorter eustachian tube, more horizontal position and with a more flaccid cartilage and low immunity, the infection is more severe in children (Bluestone and Klein 2001; Weiner and Collison 2003). The etiology and prevalence of ear infection differs with geographical areas and climate conditions (Brook and Frazier 1996; Muluye et al. 2013). However; normal flora of the skin such as *Pseudomonas aeruginosa, Staphylococcus aureus*, *Proteus mirabilis, Klebsiella pneumonia* and *Escherichia coli* that can easily enter through perforated ear have been reported as the main agents of otitis media (Abera and Kibret 2011).

Development and spread of resistant bacteria due to the over and indiscriminate use of antibiotics is a global public health threat (Spellberg et al. 2008). Due to the limited laboratory diagnosis in developing countries, physicians are often forced to syndromatic diagnosis and prescription of broad spectrum antibiotics for most infections that led to emergence of drug resistant bacterial strains (Okeke et al. 2007; Lee et al. 2012). Hence, current information on microbial resistance and the prevalence of the pathogenic bacteria needs to be available at national and local levels to guide the rational use of the existing antimicrobials. In Ethiopia, few studies reported high prevalence of ear infection and multi drug resistance to the commonly prescribed antibiotics for treatment of ear infection (Abera and Kibret 2011; Seid et al. 2013; Muluye et al. 2013; Melaku and Lulseged 1999). However, there is no published data in study area on the prevalence and antimicrobial susceptibility pattern of bacterial pathogens causing otitis media. Hence, the aim of this current study was to fill the existing knowledge gap.

## Methods

### Study design, area, specimen collection and sample size

A cross-sectional study was conducted at Ayder referral hospital, Northern Ethiopia. Patients who visited the ENT clinic of the hospital with middle ear infection/or acute otitis media from October 2014 to June 2015 were consecutively enrolled into the study. A total of 162 ear discharges samples were collected by Otorhinolaryngologist using sterile cotton swabs after getting written informed consent from each participant and their parents. Participants already on antibiotic treatment were excluded. Socio demographic and clinical data were collected using standard/ or structured questionnaire.

#### Study area

Ayder referral hospital, which is located 783 km North of Addis Ababa, is the only referral hospital in Tigray regional state with 504 beds giving services for 9 million people of Tigray, North Amhara and Afar regions. The hospital is giving tertiary level clinical service within many departments including ENT clinic.

#### Sample size determination

Sample was determined by taking the prevalence of 91.7 % from a study performed in Dessie Ethiopia (Abera and Kibret 2011 ) and with margin of error (d) 0.05 and confidence interval (Zά/2) 95 %).

### Isolation and identification of bacteria

For the detection of pathogenic bacteria, collected swabs were plated on MacConkey agar, Blood agar, Manitol Salt agar and Chocolate agar plates. MacConkey agar, blood agar and Manitol Salt agar were incubated in aerobic condition, whereas chocolate plate was kept in a candle jar, which can generate about 5 % CO_2_. All of the inoculated media were incubated at 37 °C for 18–24 h. Isolates were identified by colony morphology, Gram staining reaction, Catalase test, Coagulase test, Oxidase test, Triple Sugar Iron agar (TSI) (OXOID, UK), Citrate utilization test (BBL™), Urease test (BBL™) Motility Indole Lysine (MIL) [BBL™] and Optochin test (Cheesbrough 2006).

### Antimicrobial susceptibility testing

Disk diffusion assay was performed to assess the antibiotic resistance/susceptibility pattern of bacterial isolates. Antimicrobial susceptibility testing was carried out on Muller-Hinton agar (Oxoid, England) using the single disc diffusion technique against tetracycline (30 μg), penicilin G (10 μg), erythromycin (15 μg), gentamicin (10 μg), ciprofloxacin (5 μg), norfloxacillin (10 μg), trimethoprim-sulphamethoxazole (25 μg), nitrofurantonin (300 µg), doxycycline (30 µg), ceftriaxone (30 μg), ampicillin (10 μg) and amoxicillin clavulanic acid (10 μg) (all Oxoid, England). Selection of these antibiotics was based on the frequently used in the country for the treatment of otitis media. Results were reported as sensitive, intermediate and resistance according to Clinical Laboratory Standards Institute (CLSI 2015) guide lines. An isolate was defined as being multidrug resistant if it is resistant to three or more of the antimicrobial agents tested and based on the antimicrobial categories as stated by Magiorakos et al (2012).

### Quality control and data analysis

A standard bacteriological procedure was followed to keep the quality of all laboratory tests. American Type Culture Collection (ATCC) strains (*E. coli* ATCC 25922, *P. aeruginosa* ATCC 27853, *S.aureus* ATCC25923, *K. pneumoniae* ATCC 700603 and *P. mirabilis* ATCC 35659) were used as controls for culture and sensitivity testing. Data were entered and analyzed using SPSS version 20 software and p value of <0.05 was considered statistically significant.

### Ethical issues

The study was approved and ethically cleared by the Research and Ethical Review Committee of Mekelle University, College of Health Sciences. Written informed consent was obtained from each participants and parents or caretakers. Result finding were communicated with ENT doctor to help the patients.

## Result

Out of the total 162 patients, 105 (64.8 %) of them were males and 57 (35.2 %) females. The age range of participants was from 3 months to 69 years and mean age of (mean 21.9 12 ± 1.81 [SD]). Most participants 41 (25.3 %) were in the age group of 6–10 years, and most participants 89 (54.9 %) were from the rural areas. Chronic infection was seen among 115 (71 %) participants, and referred cases from other healthcares were 117 (72.2 %). Ninety three (57.4 %) had history of previous hospital visit and treatment. One hundred nine (67.3 %) of the patients with ear infections had decreased hearing status (Table 1).

**Table 1:** Socio demographic and clinical manifestation of patients with ear discharge at Ayder referral hospital, North Ethiopia (November 2014–June 2015)

Variable	Frequency (%) (N = 162)
Sex	
Male	105 (64.8)
Female	57 (35.2)
Age	
0–5	30 (18.5)
6–10	41 (25.3)
11–15	22 (13.6)
16–20	11 (6.8)
21–25	23 (14.2)
26–30	9 (5.5)
>30	26 (16)
Residence	
Urban	73 (45.1 %)
Rural	89 (54.9 %)
Previous hospital visit and treatment	
Yes	93 (57.4 %)
No	69 (42.6 %)
Ear involved	
Right	58 (35.8 %)
Left	35 (21.6 %)
Both	69 (42.6 %)
Hearing status	
Well	53 (32.7 %)
Decreased	109 (67.3 %)
Infection type	
Acute	47 (29 %)
Chronic	115 (71 %)
Discharge type	
White	64 (39.5 %)
Bloody	33 (20.4 %)
Yellow	38 (23.5 %)
Green	27 (16.7 %)
Reason to visit ENT clinic	
Self	45 (27.8 %)
Referred	117 (72.2 %)

**Fig. 1:**
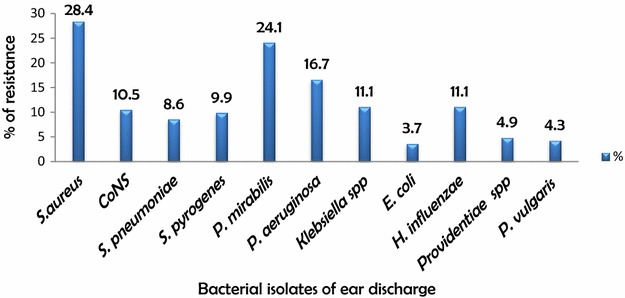
Frequencies of bacterial species isolated from ear discharges of patients attending ARH, North Ethiopia (November 2014–June 2015)

**Fig. 2:**
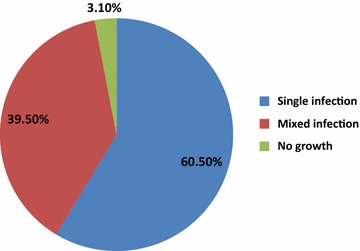
Single and mixed infection among ear discharge patients attending Ayder referral hospital North Ethiopia (November 2014–June 2015)

In this study, pathogens were isolated from 157 (98.2 %) of the patients with a total bacterial isolates of 216. *S. aureus* 46 (28.4 %), *P*. *mirabilis* 39 (24.1 %), *P. aeruginosa* 27 (16.7 %) *Klebsiella* spp. and *H. influenzae* 18 (11.1 % each) were the predominant bacterial isolate respectively. Gram negative bacteria 121 (56 %) were more dominant than gram positive 95 (43.5 %) (Fig. 1). Out of the patient samples with positive culture results, single and mixed infection was seen in 60.5 and 39.5 % respectively. Only 5 (3.1 %) patient samples showed negative culture result (Fig. 2).

The highest number of bacteria 98 (45.3 %) were isolated in the age group of 0–5 years (p = 0.02). *S. aureus, P. mirabilis, P. aeruginosa, S. pyogenes, S. pneumoniae* and *H. influenzae* were the dominant bacterial isolates in this age group. Although not statically significant, slightly more bacteria were recovered from male patients and rural resident. Chronic patients 136 (63 %) (p = 0.042) and referred patients from other heath care 132 (61.1 %) (p = 0.045) showed significantly high bacterial isolates. *S. aureus*, *P. mirabilis*, *P. aeroginosa* and *Klebsiella* spp. were the most common in chronic otitis media in this study (Table 2).

**Table 2:** Prevalence of bacterial isolates by age, sex, residence and infections type of study participants at Ayder referral hospital, North Ethiopia (Nov 2014–June 2015)

Variables	*S. aureus*	*CoNS*	*S. pyogenes*	*S. pneuniae*	*P. mirabilis*	*P. aeruginosa*	*Klebsiella* spp.	*E. coli*	*H. influenzae*	*Providentiae* spp.	*P. vulgaris*	Total (n = 216)	P value
Age													
0–5	21 (46)	7 (41)	9 (56)	11 (73.3)	19 (48.7)	17 (63)	3 (16.7)	0	9 (56.3)	0	2 (28.6)	98 (44.4)	*0.02*
6–10	6 (13)	0	2 (12.5)	1 (6.7)	9 (23.1)	5 (19)	1 (5.6)	0	3 (18.8)	2 (25)	0	29 (13.4)	
11–15	8 (17)	2 (11.8)	1 (6.3)	0	4 (10.3)	1 (3.7)	4 (22.2)	1 (16.7)	1 (6.3)	1 (12.5)	0	23 (10.7)	
16–20	4 (8.7)	1 (5.9)	3 (18.8)	2 (13.3)	3 (7.7)	0	0	2 (33.3)	1 (6.25)	3 (37.5)	2 (28.6)	21 (9.7)	
21–30	1 (2.3)	6 (35.3)	0	1 (6.7)	1 (2.6)	3 (11)	3 (16.7)	1 (16.7)	0	0	1 (14.3)	17 (7.9)	
>30	7 (15)	1 (5.9)	1 (6.3)	0	3 (7.7)	1 (3.7)	7 (38.9)	2 (33.3)	2 (12.5)	2 (25)	2 (28.6)	28 (13)	
Total	46	17	16	15	39	27	18	6	16	8	7	216	
Sex													
Male	25 (54)	6 (35)	6 (37)	9 (60)	20 (51)	13 (48)	7 (39)	2 (33)	14 (87.5)	6 (75)	4 (57)	112 (52)	0.43
Female	21 (46)	11 (65)	10 (63)	6 (40)	19 (49)	14 (52)	11 (61)	4 (67)	2 (12.5)	2 (25)	3 (43)	104 (48)	
Address													
Urban	32 (70)	9 (53)	7 (44)	4 (27)	20 (51)	8 (30)	8 (44)	1 (17)	7 (44)	4 (50)	5 (71)	106 (49)	0.42
Rural	14 (30)	7 (47)	9 (56)	11 (73)	19 (49)	19 (70)	10 (66)	5 (83)	9 (66)	4 (50)	2 (29)	110 (51)	
Infection type													
Acute	15 (33)	8 (17.4)	7 (43.8)	4 (30.8)	12 (30.8)	10 (37)	5 (27.8)	0	7 (43.8)	6 (60)	4 (57)	80 (37)	*0.042*
Chronic	31 (67)	7 (15.2)	9 (56.2)	9 (69.2)	27 (69.2)	17 (63)	13 (72.2)	6 (100)	9 (56.2)	4 (40)	3 (43)	136 (63)	
Reason to visit ENT													
Self	15 (33)	6 (38)	11 (69)	3 (13)	9 (23)	18 (67)	7 (38.9)	2 (33.3)	5 (31.3)	2 (20)	6 (66.7)	84 (38.9)	*0.045*
Referred	31 (67)	10 (62)	5 (31)	12 (87)	30 (77)	9 (33)	11 (61.1)	4 (66.7)	11 (63.7)	8 (80)	1 (14.3)	132 (61.1)	

Italic values indicate statistically significant association (P < 0.05)

In vitro antibiotic susceptibility of gram positive bacterial isolates (Table 3) was from 20 to 100 %. Of 46 *S. aureus* isolates, 100 % were resistant to ampicillin, tetracycline and penicillin (100 % each), (67.4 %) to ceftriaxone and (63 %) to doxycycline. Likewise Coagulase negative Staphylococci were resistance to trimethoprim-sulphamethoxazole and ampicillin (100 % each), (88 %) to tetracycline, and amoxicillin clavulanic acid and penicillin 13 (76.5 % each). Isolated *S. pneumoniae* showed resistance to amoxicillin clavulanic acid, doxycycline and penicillin (93 % each), tetracycline 100 %, norfloxacin and trimethoprim-sulphamethoxazole (80 % each) and ampicillin (86.7 %). High resistance, (87.5 %) and (81.3 %) was seen to trimethoprim-sulphamethoxazole and ampicillin respectively by *S. pyogenes*. Less resistance was observed to ciprofloxacin, gentamicin, erythromycin and norfloxacin by Gram positive isolates.

**Table 3:** Antimicrobial resistance pattern of bacterial isolates from ear discharge samples of study participants at Ayder referral hospital, North Ethiopia (November 2014–June 2015)

Bacterial isolates	Resistance pattern of antimicrobial agents (R %)											
	AMC	CRO	CN	DO	CIP	SXT	NOR	E	AML	T	P	F
*S. aureus* (n = 46)	28 (60.9)	31 (67.4)	19 (41.3)	29 (63)	10 (21)	31 (67.4)	20 (43.5)	18 (39)	46 (100)	46 (100)	46 (100)	NA
*CoNS* (n = 17)	13 (76.5)	4 (23.5)	6 (35.3)	11 (64.7)	9 (52.9)	17 (100)	6 (35.3)	10 (59)	17 (100)	15 (88)	13 (76.5)	NA
*S. pneumoniae* (n = 15)	14 (93)	9 (60)	3 (20)	14 (93)	3 (20)	12 (80)	12 (80)	7 (47)	13 (86.7)	15 (100)	14 (93)	NA
*S. Pyrogenes* (n = 16)	10 (62.5)	9 (56.3)	4 (25)	6 (40)	3 (20)	14 (87.5)	4 (25)	4 (25)	13 (81.3)	12 (75)	11 (68.8)	NA
*P. mirabilis* (n = 39)	26 (66.7)	14 (35.9)	7 (17.9)	25 (64)	– (0)	25 (64)	(0)	NA	25 (64)	26 (66.7)	26 (66.7)	26 (66.7)
*P. aeruginosa* (n = 27)	24 (88.9)	17 (62.3)	17 (62.3)	25 (93.6)	10 (37)	19 (70.4)	17 (62.3)	NA	27 (100)	27 (100)	26 (96.3)	27 (100)
*Klebsiella* spp. (n = 18)	13 (72.2)	8 (44.4)	7 (38.9)	11 (61.1)	2 (11)	14 (77.8)	9 (50)	NA	16 (88.9)	16 (88.9)	17 (94.4)	10 (55.6)
*E. colin* (*n* = 6)	3 (50)	4 (66.7)	1 (16.7)	5 (83.3)	1 (16.7)	4 (66.7)	1 (16.7)	NA	5 (83.3)	3 (50)	2 (33.3)	5 (83.3)
*H. influenzae* (*n* = 16)	9 (53.6)	5 (31.3)	3 (18.8)	8 (50)	2 (12.5)	9 (53.6)	4 (25)	NA	11 (68.8)	9 (53.6)	13 (81.3)	12 (68.8)
*Providentiae* spp. (*n* = 8)	6 (75)	3 (37.5)	3 (37.5)	7 (87.5)	0	8 (100)	1 (12.5)	NA	6 (75)	8 (100)	5 (62.5)	4 (500)
*P. vulgaris* (n = 7)	3 (42.9)	3 (42.9)	0	1 (12.3)	0	4 (57)	0	NA	6 (85.7)	5 (71.4)	6 (85.7)	3 (42.9)

*AMC* amoxicillin clavulanic acid, *CRO* ceftriaxone, *CN* gentamicin, *DO* doxycycline, *CIP* ciprofloxacin, *SXT* trimethoprim-sulphamethoxazole, *E* erythromycin, *OX* oxacillin, *NOR* norfloxacin, *F* nitrofurantonin, *T* tetracycline, *AML* ampicillin, *P* penicillin, *NA* not applicable

The overall antimicrobial resistance level of gram negative bacteria was from 0 to 100 %. *P. aeruginosa* was resistant to tetracycline, ampicillin, nitrofurantonin (100 % each), 96.3 % to penicillin and amoxicillin clavulanic acid (88.9 %). *K. pneumoniae* were high resistant to penicillin (94.4 %), ampicillin and tetracycline (88.9 % each). Similarly, *E. coli* showed 50 % resistance to doxycycline, and ampicillin and nitrofurantonin (83.3 % each). *P. mirabilis* were resistant to amoxicillin clavulanic acid, tetracycline, penicillin and nitrofurantonin (66.7 % each), but all isolates were sensitive to ciprofloxacin and norfloxacin. *H. influenzae* were (81.1 %) resistant to penicillin. Ciprofloxacin, norfloxacin, gentamicin, ceftriaxone and amoxicillin clavulanic acid were effective against most gram negative bacteria isolates.

Anti biogram drug resistance pattern of isolates showed that 71.1, 70.6, 80 and 75 % of *S. aureus*, CoNS, *S. pyogenes* and *S. pneumoniae* showed multi drug resistance respectively, with an overall gram positive multi drug resistance rate of 73.4 %. On the other hand, 84.6, 85.2, 76.5, 66.7, 62.5 and 71.4 % of isolated *P. mirabilis, P. aeruginosa, Klebsiella* spp., *E. coli, H. influenzae, Providentiae* spp. and *P. vulgaris* were multidrug resistant respectively with overall gram negative MRD rate of 78.3 %. In this study 21 (9.7 %), 14 (6.5 %), 8 (3.7 %) and 4 (0.9 %) of the isolates were resistant to 7, 8, 9, and 10 antibiotics tested in this study. However, none of the isolates were sensitive to all antibiotics tested. In general the multi drug resistance rate of in this study was seen in (74.5 %) of the isolates (Table 4).

**Table 4:** Multiple drug resistance patterns of gram positive and gram negative bacteria from ear discharge samples of study participants at Ayder referral hospital, North Ethiopia (November 2014–June 2015)

Organisms	Antibiogram pattern No (%)									
	R1	R2	R3	R4	R5	R6	R7	R8	R9	R10
*S. aureus* (46)	7 (15.2)	6 (13)	6 (13)	10 (21.7)	5 (10.9)	3 (6.5)	5 (10.9)	2 (4.3)	1 (2.2)	1 (2.2)
CoNS (17)	3 (17.6)	2 (11.8)	3 (17.6)	2 (11.8)	3 (17.6)	2 (11.8)	1 (5.9)	–	1 (5.9)	–
*S. pneumoniae* (15)	3 (20)	1 (6.7)	3 (20)	2 (13.3)	5 (33.3)	1 (6.7)	1 (6.7)	–	–	–
*S. Pyogenes* (16)	1 (6.3)	3 (18.8)	3 (18.8)	2 (12.5)	–	2 (12.5)	2 (12.5)	2 (12.5)	–	1 (6.3)
*P. mirabilis* (39)	2 (5.1)	4 (10.3)	6 (15.4)	7 (20)	3 (7.7)	6 (15.4)	5 (12.8)	3 (7.7)	2 (5.1)	1 (2.6)
*P. aeruginosa* (27)	1 (3.7)	3 (11.1)	4 (14.8)	5 (18.5)	5 (18.5)	1 (3.7)	2 (7.4)	3 (11.1)	2 (7.4)	1 (3.7)
*Klebsiella* spp. (17)	2 (18)	2 (18.2)	3 (17.7)	5 (29.4)	3 (17.7)	–	1 (5.9)	1 (5.9)	–	–
*E. coli* (6)	2 (33.)	1 (16.7)	1 (16.7)	1 (16.7)	1 (16.7)	1 (16.7)	–	–	–	–
*H. influenzae* (16)	3 (18.8)	5 (31.3)	1 (6.3)	2 (12.5)	2 (12.5)	2 (12.5)	3 (18.8)		–	–
*Providentiae* spp. (8)	–	2 (25)	1 (12.5)	2 (25)	1 (12.5)	1 (12.5)	1 (12.5)		–	–
*P. vulgaris* (7)	1 (14)	1 (14)	1 (14.3)	2 (28.6)	1 (14.3)	1 (14.6)	–	–	–	–
Total (216)	25 (11.6)	33 (15.3)	32 (14.8	40 (18.5)	27 (12.5)	20 (9.3)	21 (9.7)	14 (6.5)	8 (3.7)	4 (1.9)

R1, R2, R3, R4, R5, R6, R7, R8, R9, R10 stands for resistance of the isolates for one, two, three, four, five, six, seven, eight, nine and ten antibiotics tested in this study, respectively

*CoNS* coagulase negative *staphylococci*

## Discussion

In this study the prevalence of bacteria among OM patients was 98.2 %. This was in tandem with reports from other parts of Ethiopia 91.7 % (Abera and Kibret 2011), 89.4 % (Seid et al. 2013), 89.5 % (Muluye et al. 2013), 100 % (Diriba et al. 2004) and Nigeria, 81.9 % (Osazuwa et al. 2011). Gram-negative bacteria, 56 % were the dominant isolates of the discharging ears compared to gram- positive bacteria. Similar reports were seen from Gonder 56.4 % (Muluye et al. 2013), Dessie 74.2 % (Abera and Kibret 2011), Addis Ababa 60.5 % (Ferede et al. 2001) and Nigeria 75 % ( Iseh and Adegbite 2004) though the proportion varies.

*Staphylococcus aureus*, *P. mirabilis* and *P. aeruginosa* were the most dominant isolates in this study. This was in line with finding from Addis Ababa (Ferede et al. 2001). In contrast to ours, *Proteus* spp., *S. aureus* and *Pseudomonas* spp. were the predominant bacteria by other researchers (Abera and Kibret 2011; Muluye et al. 2013; Seid et al 2013; Melaku and Lulseged 1999; Diriba et al. 2004; Ferede et al. 2001; Abera and Biadglegne 2009; Yismaw et al. 2010).

Report from Cote D’Ivoire have also showed *P. aeruginosa* and *S. pneumoniae* to be the leading isolates (Tanon-Anoh et al. 2006), *H. influenzae*, *S. pneumoniae* and M. Catarrhalis from Brazil (Pereira et al.^ 2004^**)**, and *S. pneumoniae* and *H. influenzae* from Israel (Sakran et al., 2006) were the dominant isolates. *P*. *aeruginosa,* the third dominant cause of OM in this study was reported in very low prevalence in from Gondar (Yismaw et al. 2010). However, other researchers have reported *P. aeruginosa* and *S. aureus* as the most dominant cause of OM (Iseh and Adegbite 2004; Weckwerth et al. 2009; Aslam et al. 2004). Variation in climatic and geographic could be the possible reasons for the difference in distribution of the bacteria.

Under 5 years were significantly colonized by bacterial (p = 0.02), which corroborates to results from Ethiopia (Ferede et al. 2001) and Nigeria (Iseh and Adegbite 2004). Low immune status, shorter and horizontal nature of their Eustachian tubes, frequent exposure to upper respiratory tract infections and malnutrition could be the possible justifications for the high infection in these age group (Melaku and Lulseged 1999).

There was no statistically significant association between bacteria and gender in this current study. This observation agrees well with reports from other researchers (Abera and Kibret 2011; Osazuwa et al. 2011). Unlike to this result, studies from Ethiopia (Muluye et al. 2013) and Nigeria (Egbe et al. 2010) showed that males were more infected than females, but according to the report of Hassan and Adeyemi (2007), females were more affected by ear infections.

In our study monoclonal infection was seen in 60.5 % of the patients. This observation was supported by other researchers elsewhere in the world (Shyamla and Reddy 2012; Osazuwa et al. 2011; Mansoor et al. 2009; Loy et al. 2002). A study from Iran (Ettehad et al. 2006) has reported 100 % monoclonal infection. Other researchers however, found poly microbial infection more prominent in OM (Nwokoye et al. 2012; Rao and Bhaskaran 1984). Predominant bacterial etiology of chronic OM in this study was *S. aureus* and this observation was in line with studies Iran (Ettehad et al. 2006) and India (Singh et al. 2012; Prakash et al. 2013). In contrast to this, other studies from other parts of India (Kumar and Seth 2011), Nigeria (Osazuwa et al. 2011) and Pakistan (Mansoor et al. 2009) showed different trends as *Pseudomonas* spp. being the most prevalent organism in COM which could be due to the variation in micro-organisms in different regions and effect of climate.

Prevalence of coliforms bacteria such as *K. pneumoniae* and *E. coli* in this study was 11.1 and 3.7 % respectively. This result was tandem to reports by Prakash et al.9.42 and 7.33 %, (Prakash et al. 2013) and Mansoor et al. 8 and 4 %, whereas Poorey and lyer (Mansoor et al. 2009) have reported a high-incidence 25.4 % for *Klebsiella* spp. A recent study by Shyamala and Reddy (2012) from India showed a little different trend than our result, where *E. coli* was reported in 12 % and *Klebsiella* spp. in 5 % of cases. Isolation of fecal bacteria like *E. coli*, *Klebsiella* spp. and water bacteria like *Pseudomonas* spp. may indicate that individuals are at risk of infection due to poor hygiene conditions.

In vitro antimicrobial susceptibility pattern revealed that isolates were highly resistant to most antibiotics. *S. aureus* were 100 % resistant to Penicillin, Tetracycline and Ampicillin. This result was in line with that of study done in other parts (Osazuwa et al. 2011) who reported 100 % for ampicillin and tetracycline and in Ethiopia where 93 % penicillin, 86 % ampicillin and 79 % for tetracycline was reported (Ferede et al. 2001). This was however, higher than other findings 65 % for tetracycline (Abera and Kibret 2011), 79 % ampicillin (Prakash et al. 2013), 81.8, 52.35 and 90.9 % for ampicillin, tetracycline and penicillin respectively (Seid et al. 2013), 46 % for tetracycline, 48 % ampicillin, and 50 % penicillin (Muluye et al. 2013) and 76 % ampicillin (Osazuwa et al. 2011). However *S. aureus* isolates were less resistant for gentamicin, ciprofloxacin, norfloxacillin and erythromycin which was similar with the results of other researches from elsewhere (Abera and Kibret 2011; Seid et al. 2013; Muluye et al. 2013) for gentamicin and ciprofloxacin, and for gentamicin (Osazuwa et al. 2011).

Isolated CoNS showed 100 % resistant for trimethoprim-sulphamethoxazole and ampicillin, and 88 % for tetracycline. This was in tandem to reports from India (Prakash et al. 2013). But our result was higher than reports from other places in Ethiopia (Muluye et al. 2013), where 39, 47.8 and 47.8 % resistance was reported for ampicillin, trimethoprim-sulphamethoxazole and tetracycline respectively. CoNS were however, sensitive to ceftriaxone, gentamicin and norfloxacin which is in line with Muluye et al. (2013) from Gonder. Unlike other isolates, higher resistance to Ciprofloxacin and Erythromycin was seen by CoNS in contrast to the study conducted from Iran (Pereira et al. 2004). Over all, gram positive bacteria in this study showed different resistance pattern ranging from 20 to 100 %.

*Pseudomonas aeruginosa* was the most resistant isolate to many antibiotics in this study, which is in agreement with other researcher (Abera and Kibret 2011; Prakash et al. 2013). Resistance for tetracycline, nitrofurantonin and ampicillin was 100 %; similar result was reported from other parts (Osazuwa et al. 2011). Relatively, low resistance 84 % for tetracycline (Abera and Kibret 2011), 61 % ampicillin (Osazuwa et al. 2011) was reported from other researchers.

However; it was sensitive to Ciprofloxacin which in lines with other studies from elsewhere (Abera and Kibret 2011; Seid et al. 2013; Muluye et al. 2013; Yismaw et al. 2010; Weckwerth et al. 2009; Rao and Bhaskaran 1984; Osazuwa et al. 2011; Prakash et al. 2013). *Pneumoniae* was 94 % resistant for penicillin and 78.9 % for both ampicillin and tetracycline. This was comparable to reports for other parts of the world (Seid et al. 2013; Muluye et al. 2013; Osazuwa et al. 2011). Less resistance for tetracycline and penicillin was obtained from Ethiopia (Muluye et al. 2013). Gentamicin and Ciprofloxacin were the most effective antibiotics against K*. pneumoniae* in this study.

The second most prevalent isolates *P. mirabilis* was relatively less resistant to the antibiotics compared to other isolates. Yet, 66.7 % resistance was seen to amoxicillin clavunilic acid, tetracycline, penicillin and nitrofurantonin, which corroborates other findings (Abera and Kibret 2011; Seid et al. 2013; Muluye et al. 2013; Prakash et al. 2013). All isolates of *P. mirabilis* were 100 % sensitive to ciprofloxacin and norfloxacillin, and less resistance was seen for gentamicin and ceftriaxone as well.

Antibiograms pattern of the isolates revealed that multidrug resistance was quite high; which may result in treatment failure and disease complications of OM. Some of the isolates were resistant for nearly all the antimicrobial drugs tested. This was in agreement with (Seid et al. 2013; Muluye et al. 2013; Prakash et al. 2013). MDR rate for gram positive and gram negative was from 71.1 to 80 % and 66.7 to 85.2 % respectively. This high drug resistance might reflect the degree of misuse of antibiotics, which is a global problem mainly through their purchase without prescription in the local pharmacies and drug stores and through inappropriate prescribing habits and an over-zealous desire to treat every infection (Seid et al. 2013).

Over all, the MDR rate in this study was 74.5 % which is high than others studies and this could be due to the fact that our patients were 81.4 and 57.4 % referral and had treatment previously for the same case respectively. In addition to this most of the bacteria isolated in this study were biofilm formers which are 10–1000 times or more resistant to antibiotic treatment when compared with genetically identical planktonic bacteria (Diriba et al. 2004). Unavailability of culture facilities and empirical prescription of these antimicrobial drugs and negligence on patient part might also be the important contributing factor for the development of multidrug resistance.

In conclusion, bacterial isolates in this present study was so high especially in under five children. Most of the patients had hearing problem. *S. aureus P. mirabilis* and *P. aeruginosa* were the dominant isolates in OM. Most of the isolates showed very high levels of antimicrobial resistance. However, ciprofloxacin, gentamicin, norfloxacin and erythromycin were effective against most of the bacterial isolates, and can be used in the treatment of OM. Hence appropriate and early treatment of ear infection using culture and susceptibility testes can play great role in management of otitis media and prevent further emerging of multi drug-resistant bacteria.

## Limitation

The study did not isolate strict anaerobic bacteria and fungi which are also the causative agents for OM.
